# Supplementary figures and images for: Oligosaccharide production and signaling correlate with delayed flowering in an Arabidopsis genotype grown and selected in high [CO2]
Source: PLoS One. 2023 Dec 28;18(12):e0287943. doi: 10.1371/journal.pone.0287943 (PMC10754469; doi:10.1371/journal.pone.0287943)

S1 Figure

(a)

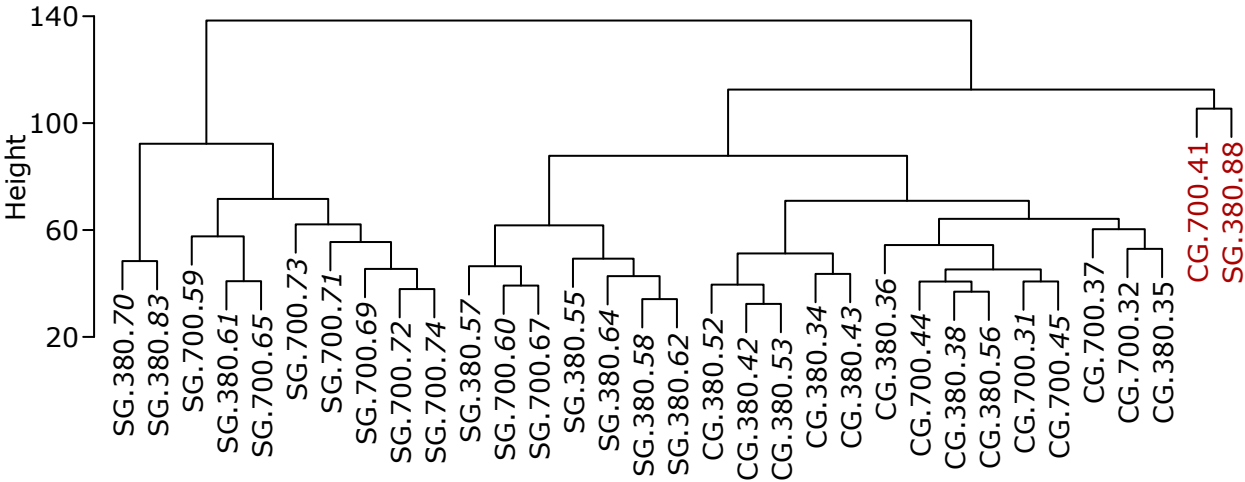

(b)

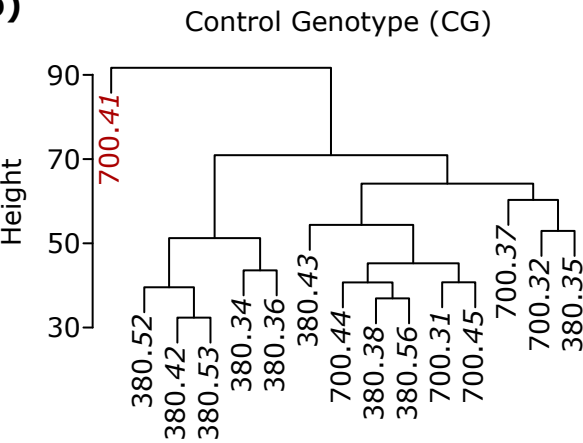

(c)

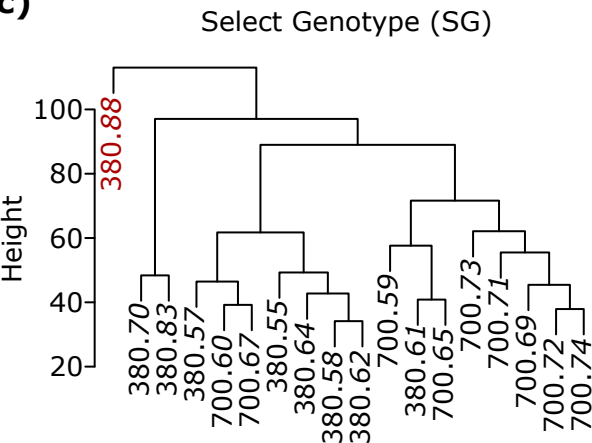

Supplement: S1 Fig — Outliers shown in red. These were removed for subsequent analyses. Sample labels include strain (SG or CG), treatment [CO2] (380 or 700 ppm), and sample identification number. (PDF) [file pone.0287943.s001.pdf]

S2 Figure

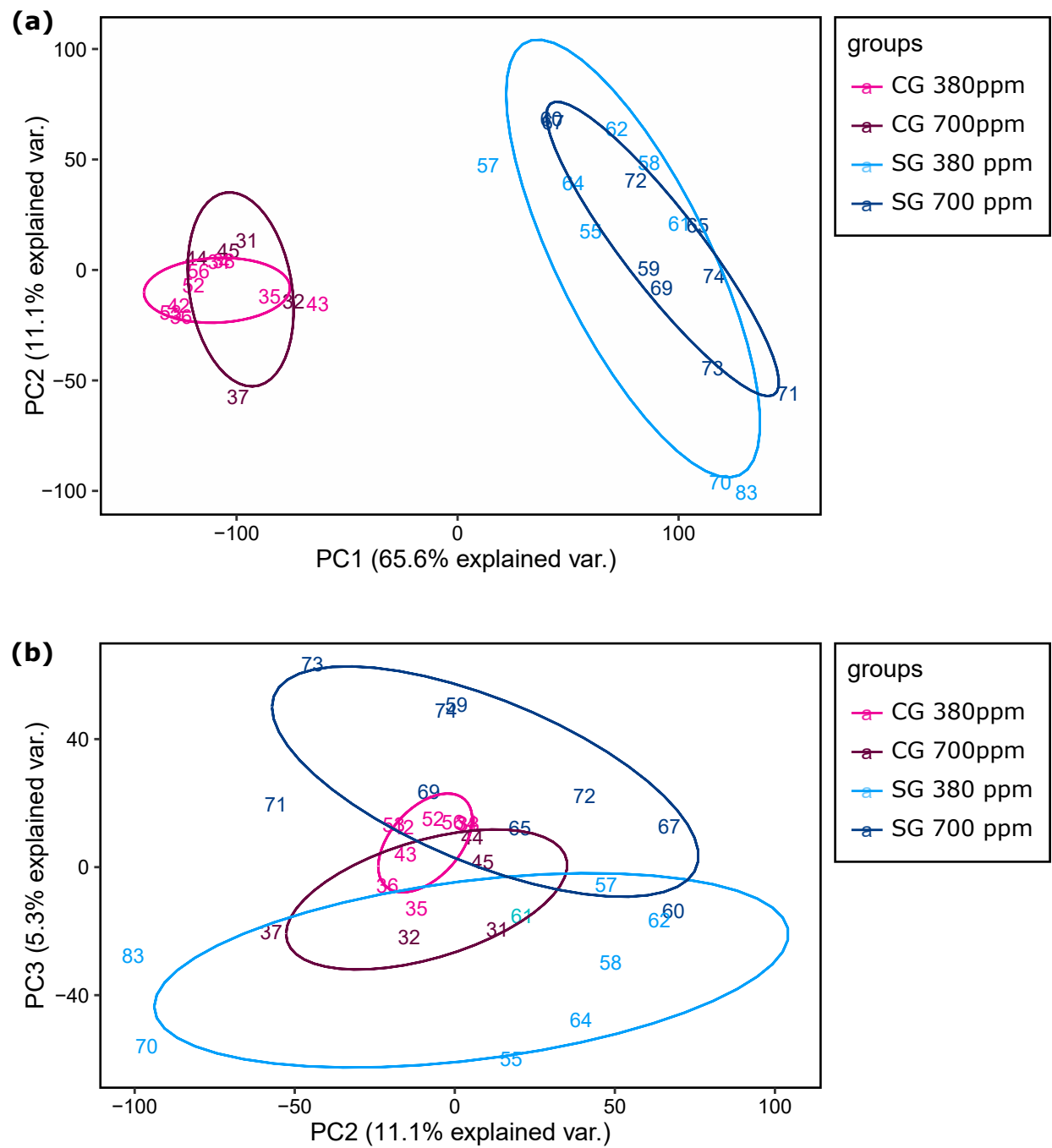

Supplement: S2 Fig — Samples grouped by genotype and [CO2]. (PDF) [file pone.0287943.s002.pdf]
