# Supplementary material for: Oligosaccharide production and signaling correlate with delayed flowering in an Arabidopsis genotype grown and selected in high [CO2]
Source: PLoS One. 2023 Dec 28;18(12):e0287943. doi: 10.1371/journal.pone.0287943 (PMC10754469; doi:10.1371/journal.pone.0287943)
Supplement: S2 Table — Table S2a is an expanded version of Table 2 in the main text, including all Functional Annotation Clusters with Enrichment Scores greater than 1.3. Tables S2b-h are full functional annotation outputs from DAVID for each comparison. (ZIP) [file pone.0287943.s004.zip › S2_Table.pdf]

S2 Table METADATA. Full functional annotation tables (outputs from DAVID) for each comparison stored as .txt and .pdf files. S2a Table provides is an expanded version of Table 2 in the main text including all Functional Annotation Clusters with Enrichment Scores greater than 1.3. S2b through S2h Tables are the full Functional Annotation Cluster outputs. Comparisons are between the CG and SG genotypes (S2b and c, file names including 'GENO'), between current and elevated CO_2 levels across both genotypes (S2d, file name including 'CO2'), and between current and elevated CO_2 levels within each genotype (S2e-h, file names including 'CG' or 'SG' and 'CO2'). Files are separated into an increase from the control (files ending in '_effect', or a decrease from the control (files ending in '_effectN'). *(Files are included as separate PDF or tab delimited (.txt) files named S2a_Table, S2b_Table_DAVID_GENO_sig_effect, S2c_Table_DAVID_GENO_sig_effectN, S2d_Table_DAVID_CO2_sig_effectN, S2e_Table_DAVID_CG_CO2_sig_effect, S2f_Table_DAVID_CG_CO2_sig_effectN, S2g_Table_DAVID_SG_CO2_sig_effect, S2h_Table_DAVID_SG_CO2_sig_effectN.)*

S2a Table is the summary table of the top 15 significant clusters in each comparison; the top 5 are shown in Table 2 in the main text.

S2b-h Table are the outputs from DAVID (tab delimited). Input list being transcript identifiers having enrichment scores +/- 1, analyzed relative to default *Arabidopsis thaliana* reference.

S2 Table KEY for DAVID output files:

Enrichment Score = Obtained by taking the -log10 of each p-value, then averaging the results for each result in the cluster.

Each cluster contains annotation terms deemed to be related based on similarity of transcript IDs used to call those annotations.

Category = database against which input list was referenced.

Term = functional annotation term.

Count = number of genes from input list associated with the functional annotation term being tested; % = percent of total genes in input list.

P-Value = EASE score, derived from a modified Fisher exact test (https://david.ncifcrf.gov/helps/functional_annotation.html#E3)

Genes = list of gene IDs associated with that functional annotation term.

List Total = Number of input gene IDs that are members of the functional annotation term being tested.

Pop Hits = Total number of genes in background that are members of the functional annotation term being tested.

Pop Total = Background: all possible genes that could be measured.

Fold Enrichment = Calculation of the increase in the fraction of genes that are members of the functional annotation term over the expected fraction that would be expected by chance. Equal to (Count/List Total)/(Pop Hits/Pop Total)

Bonferroni, Benjamini, FDR = P-value adjustments for multiple comparisons.
