# Supplementary material for: Oligosaccharide production and signaling correlate with delayed flowering in an Arabidopsis genotype grown and selected in high [CO2]
Source: PLoS One. 2023 Dec 28;18(12):e0287943. doi: 10.1371/journal.pone.0287943 (PMC10754469; doi:10.1371/journal.pone.0287943)
Supplement: S2 Table — Table S2a is an expanded version of Table 2 in the main text, including all Functional Annotation Clusters with Enrichment Scores greater than 1.3. Tables S2b-h are full functional annotation outputs from DAVID for each comparison. (ZIP) [file pone.0287943.s004.zip › S2a_Table.pdf]

| Comparison | Functional Annotation Clusters | Enrich. Score | Unique gene IDs: |
|---|---|---|---|
| **Genotype** | Serine/threonine and protein kinase activity | 21.43 | 1573 |
| *Increase from CG to SG* | Transmembrane or membrane components | 11.93 | |
| | Calcium-binding region, serine/threonine kinase, peptidyl-serine phosphorylationsignal transduction | 7.5 | |
| | ADP and DNA binding, leucine-rich repeats | 5.68 | |
| | Ankyrin repeat and PGG domains | 5.55 | |
| | Magnesium ion binding, phospholipid-transporting P-type ATPase activity | 4.22 | |
| | S-receptor-like serine/threonine-protein kinase, S-locus glycoprotein, carbohydrate binding, lectin | 3.3 | |
| | Cytoplasmic and extracellular topological domain, glycoprotein, N-linked glycosylation site, signal peptide | 3.2 | |
| | Endocytosis, clathrin binding, clathrin coat, cytoplasmic vesicle | 3.13 | |
| | MAP kinase site/activity | 2.87 | |
| | Manganese binding, Protein Phosphatase 2C (PP2C), serine/threonine phosphatase activity | 2.84 | |
| | ARM repeat, Armadillo-like helical/fold | 2.5 | |
| | Golgi transport complex, clathrin-coated vesicle membrain, EGF-like calcium binding, protease-associated | 2.32 | |
| | EGF-like calcium binding, aspartate/asparagine hydroxylation, wall-associated receptor kinase galacturonan-binding domain, polysaccharide binding | 2.22 | |
| | Leucine-rice repeat | 2.1 | |
| **Genotype** | Chloroplast | 172.46 | 1987 |
| *Decrease from CG to SG* | Ribosome | 37.42 | |
| | Chloroplast thylakoid lumen | 19.91 | |
| | Ribosomal RNA-binding | 9.38 | |
| | Llipid biosynthesis and metabolism | 6.11 | |
| | Flavin adenine dinucleotide (FAD) binding | 6.06 | |
| | Photorespiration, mitochondrial membrane | 4.33 | |
| | PSII oxygen evolving complex, calcium ion binding | 4.14 | |
| | Translation and ribosome | 3.88 | |

| | | | |
|---|---|---|---|
| | Heat shock/unfolded protein binding | 3.58 | |
| | Redox/thioredoxin, disulfide oxidoreductase activity | 3.05 | |
| | PSI, rubisco, carbon fixation and light harvesting | 3.01 | |
| | Membrane | 2.78 | |
| | PSI, PSII, Chlorophyll a/b, antenna protein | 2.66 | |
| | Cyclophilin-type peptidyl-prolyl cis-trans isomerase | 2.64 | |
| **CG** | Transit peptide, chloroplast thylakoid membrane | 20.13 | 701 |
| *Increase from 380 to 700 ppm* | PSI, PSII, chlrophyll a/b, chloroplast, magnesium binding | 12.17 | |
| | Membrane, transmembrane | 5.89 | |
| | Glycolysis/gluconeogenesis, biosynthesis of amino acids/antibiotics | 4.38 | |
| | Cytochrome b5 heme-binding | 3.87 | |
| | Heme/iron binding, cytochrome P450, oxidoreductase activity, secondary metabolite biosynthesis, defense | 2.8 | |
| | Thioredoxin-like, glutathione S-transferase, posttranslational modifaction, protein turnover, chaperones | 2.68 | |
| | Tricarboxylic acid (TCA) cycle, magnesium or manganese binding, isocitrate/isopropylmalate dehydrogenase | 2.63 | |
| | Sulfer metabolism, cystein and cellular amino acid biosynthesis | 2.51 | |
| | Redox-active center, thioredoxin, oxidoreductase activity, sulfate assimilation | 2.24 | |
| | Hydrogren ion transmembrane tansport, proton-transporting ATPase activity | 2.23 | |
| | Aconitase/isopropylmalate dehydratase | 2.22 | |
| | Ferridoxin reductase-type/oxidoreductase FAD/NADP-binding, riboflavin synthase-like beta-varrel, FAD | 2.12 | |
| | Fructose-bisphosphate aldolase, phentose-phosphate pathway, methylation, fructose and mannose metabolism | 1.97 | |
| | Fatty acid metabolism | 1.74 | |
| **CG** | ATP and nucleotide binding | 4.93 | 292 |
| *Decrease from 380 to 700 ppm* | Microtubule motor protein activity | 4.39 | |
| | Nucleus, sequence-specific DNA binding, and transcription regulation | 4.35 | |
| | Cell division and mitosis | 3.55 | |
| | Zinc and metal binding | 2.76 | |

| | | | |
|---|---|---|---|
| | Meiosis, DNA repair | 2.62 | |
| | Actin, myosin, filament and motor protein | 1.65 | |
| | RNA recognition and binding | 1.63 | |
| | Nuclear pore, RNA export from and input to nucleus | 1.51 | |
| | mRNA transport, nuclear pore | 1.49 | |
| | Zinc finger, PHD-type | 1.39 | |
| | **No more significant** | | |
| **SG** | Golgi related and glycosyl and hexosyl transferase activity | 3.75 | 226 |
| *Increase from 380 to 700 ppm* | Small GTP binding and GTPase-mediated signal transduction | 2.83 | |
| | Cytoskeleton and microtubule | 2.71 | |
| | Cell wall organization | 2.24 | |
| | IQ motif and calmodulin binding | 1.79 | |
| | Copper ion binding, multicopper oxidase type 1-3 associated | 1.46 | |
| | Hydrolyzing O-glycosyl compounds, polysacharide binding, anchored component of plasma membrane | 1.4 | |
| | Kinasin motor domain, microtubule binding | 1.35 | |
| | **No more significant** | | |
| **SG** | Chloroplast and transit peptide | 82.03 | 1017 |
| *Decrease from 380 to 700 ppm* | Carbon metabolism and fixation, biosynthesis and metabolic pathways | 5.42 | |
| | Thylakoid, ATP- and metallo-peptidase activity, photoinhibition and PSII repair and catabolic processes | 4.92 | |
| | ATP-dependent peptidase activity and PUA-like domain | 4.55 | |
| | Transmembrane components | 4.21 | |
| | Potassium ion transmembrane transport | 3.23 | |
| | Iron-sulfer cluster binding | 2.88 | |
| | Starch, glycogen, sucrose metabolism and biosynthesis | 2.8 | |
| | Quinone binding, NADPH dehydrogenase complex | 2.66 | |
| | Carotenoid biosynthesis | 2.65 | |
| | PDZ domain, serine-type peptidase activity | 2.49 | |

| | |
|---|---|
| Starch and amylopectin biosynthesis, glycoside hydrolase (O-glycosyl compounds), alpha-amylase activity | 2.43 |
| Glycolysis, gluconeogenesis | 2.35 |
| Starch synthase, amyloplast, ADP-glucose synthase | 2.24 |
| FAD/NADP binding, pyridine nucleotide-disulphide oxidoreductase | 2.23 |
