# Supplementary material for: Oligosaccharide production and signaling correlate with delayed flowering in an Arabidopsis genotype grown and selected in high [CO2]
Source: PLoS One. 2023 Dec 28;18(12):e0287943. doi: 10.1371/journal.pone.0287943 (PMC10754469; doi:10.1371/journal.pone.0287943)
Supplement: S3 Table — (PDF) [file pone.0287943.s005.pdf]

S3 Table

CG	Sum	Sq	Df	F	*P*	*P* adj.
**D glucose**	**[CO_2]**	0.3672	1	3.3115	0.0776	0.1397
	**Replicate**	14.3397	1	129.3036	0.0000	**0.0000**
	**Residuals**	3.7706	34			
**fructose**	**[CO_2]**	0.563	1	3.8646	0.0575	0.1188
	**Replicate**	18.6834	1	128.237	0.0000	**0.0000**
	**Residuals**	4.9536	34			
**sucrose**	**[CO_2]**	0.888	1	1.0574	0.3111	0.3999
	**Replicate**	36.221	1	43.1498	0.0000	***0.0000***
	**Residuals**	28.541	34			
**D glucose 6 phosphate**	**[CO_2]**	1.0491	1	7.4435	0.0100	***0.0341***
	**Replicate**	17.3388	1	123.0236	0.0000	***0.0000***
	**Residuals**	4.7919	34			
**succinic acid**	**[CO_2]**	0.8764	1	12.123	0.0014	***0.0125***
	**Replicate**	5.2399	1	72.485	0.0000	***0.0000***
	**Residuals**	2.4579	34			
**L glutamine**	**[CO_2]**	0.049	1	0.4564	0.5039	0.5591
	**Replicate**	134.313	1	1253.026	<2e-16	***0.0000***
	**Residuals**	3.645	34			
**trehalose**	**[CO_2]**	0.905	1	7.2495	0.0109	***0.0341***
	**Replicate**	76.912	1	615.9084	<2e-16	***0.0000***
	**Residuals**	4.246	34			
**aspartic acid**	**[CO_2]**	2.8661	1	8.8586	0.0053	***0.0321***
	**Replicate**	4.0192	1	12.4223	0.0012	***0.0016***
	**Residuals**	11.0005	34			
**DL isoleucine**	**[CO_2]**	0.18	1	5.2082	0.0289	0.0742
	**Replicate**	324.21	1	9386.602	<2e-16	***0.0000***
	**Residuals**	1.17	34			
**glycerol**	**[CO_2]**	0.153	1	0.7015	0.4081	0.4897
	**Replicate**	250.81	1	1146.76	<2e-16	***0.0000***
	**Residuals**	7.436	34			
**glycine**	**[CO_2]**	2.4642	1	25.5551	0.0000	***0.0003***
	**Replicate**	0.0011	1	0.0117	0.9144	0.9144
	**Residuals**	3.2786	34			
**L alanine**	**[CO_2]**	0.0637	1	0.1983	0.6589	0.6589
	**Replicate**	0.7698	1	2.3962	0.1309	0.1472
	**Residuals**	10.9229	34			
**L ascorbic acid**	**[CO_2]**	1.378	1	3.8048	0.0594	0.1188
	**Replicate**	19.86	1	54.8378	0.0000	***0.0000***
	**Residuals**	12.314	34			
**L phenylalanine**	**[CO_2]**	0.1098	1	1.077	0.3067	0.3999
	**Replicate**	1.3905	1	13.644	0.0008	***0.0011***
	**Residuals**	3.4651	34			
**L serine**	**[CO_2]**	0.363	1	3.0779	0.0884	0.1446
	**Replicate**	0.045	1	0.3815	0.5409	0.5727

	**Residuals**	4.0094	34			
**L threonine**	**[CO_2]**	0.31615	1	7.1636	0.0114	***0.0341***
	**Replicate**	1.33478	1	30.2448	0.0000	***0.0000***
	**Residuals**	1.50051	34			
**L tyrosine**	**[CO_2]**	0.1185	1	0.4064	0.5281	0.5591
	**Replicate**	12.7155	1	43.6029	0.0000	***0.0000***
	**Residuals**	9.9151	34			
**L valine**	**[CO_2]**	0.3237	1	1.1002	0.3016	0.3999
	**Replicate**	2.2756	1	7.7352	0.0088	***0.0105***
	**Residuals**	10.0022	34			

**SG**	**Sum**	**Sq**	**Df**	**F**	***P***	***P* adj.**
**D glucose**	**[CO_2]**	1.2567	1	14.93	0.0005	***0.0055***
	**Replicate**	7.2508	1	86.141	0.0000	***0.0000***
	**Residuals**	2.6094	31			
**fructose**	**[CO_2]**	1.9692	1	9.1006	0.0051	***0.0228***
	**Replicate**	22.3739	1	103.4007	0.0000	***0.0000***
	**Residuals**	6.7078	31			
**sucrose**	**[CO_2]**	0.10048	1	2.8984	0.0987	0.1366
	**Replicate**	1.17604	1	33.9246	0.0000	***0.0000***
	**Residuals**	1.07465	31			
**D glucose 6 phosphate**	**[CO_2]**	0.068	1	0.3288	0.5705	0.5954
	**Replicate**	13.7722	1	66.5702	0.0000	***0.0000***
	**Residuals**	6.4134	31			
**succinic acid**	**[CO_2]**	1.8357	1	14.522	0.0006	***0.0055***
	**Replicate**	7.606	1	60.172	0.0000	***0.0000***
	**Residuals**	3.9186	31			
**L glutamine**	**[CO_2]**	1.023	1	8.3062	0.0071	***0.0256***
	**Replicate**	95.891	1	778.4801	<2e-16	***0.0000***
	**Residuals**	3.818	31			
**trehalose**	**[CO_2]**	0.991	1	7.0446	0.0124	***0.0373***
	**Replicate**	52.157	1	370.7502	<2e-16	***0.0000***
	**Residuals**	4.361	31			
**aspartic acid**	**[CO_2]**	0.8236	1	4.3683	0.0449	0.0894
	**Replicate**	0.0803	1	0.4261	0.5187	0.5187
	**Residuals**	5.8449	31			
**DL isoleucine**	**[CO_2]**	0.009	1	0.2879	0.5954	0.5954
	**Replicate**	286.362	1	8754.693	<2e-16	***0.0000***
	**Residuals**	1.014	31			
**glycerol**	**[CO_2]**	0.431	1	2.557	0.1200	0.1439
	**Replicate**	190.209	1	1127.781	<2e-16	***0.0000***
	**Residuals**	5.228	31			
**glycine**	**[CO_2]**	3.3683	1	4.172	0.0497	0.0894
	**Replicate**	21.5707	1	26.718	0.0000	***0.0000***
	**Residuals**	25.0282	31			

**L alanine**	**[CO_2]**	0.8698	1	4.986	0.0329	0.0846
	**Replicate**	3.1308	1	17.947	0.0002	***0.0002***
	**Residuals**	5.4078	31			
**L ascorbic acid**	**[CO_2]**	0.8311	1	3.4986	0.0709	0.1160
	**Replicate**	11.0261	1	46.4151	0.0000	***0.0000***
	**Residuals**	7.3642	31			
**L phenylalanine**	**[CO_2]**	0.13588	1	2.6359	0.1146	0.1439
	**Replicate**	0.05831	1	1.1312	0.2957	0.3327
	**Residuals**	1.59809	31			
**L serine**	**[CO_2]**	0.4777	1	9.5687	0.0042	***0.0228***
	**Replicate**	5.3524	1	107.2215	0.0000	***0.0000***
	**Residuals**	1.5475	31			
**L threonine**	**[CO_2]**	0.1581	1	4.3729	0.0448	0.0894
	**Replicate**	4.8651	1	134.5571	0.0000	***0.0000***
	**Residuals**	1.1208	31			
**L tyrosine**	**[CO_2]**	39.29	1	3.175	0.0846	0.1269
	**Replicate**	120.41	1	9.7302	0.0039	***0.0047***
	**Residuals**	383.64	31			
**L valine**	**[CO_2]**	0.2794	1	0.9882	0.3279	0.3689
	**Replicate**	0.1401	1	0.4955	0.4867	0.5154
	**Residuals**	8.7638	31			
