# Supplementary material for: Oligosaccharide production and signaling correlate with delayed flowering in an Arabidopsis genotype grown and selected in high [CO2]
Source: PLoS One. 2023 Dec 28;18(12):e0287943. doi: 10.1371/journal.pone.0287943 (PMC10754469; doi:10.1371/journal.pone.0287943)
Supplement: S4 Table — Whether genes were present in transcript dataset is indicated. (PDF) [file pone.0287943.s006.pdf]

**Name**	**Gene_ID**	**In_data_set**
LHY	AT1G01060	NO
CCA1	AT2G46830	YES
TOC1	AT5G61380	YES
PRR5	AT5G24470	YES
PRR7	AT5G02810	YES
PRR9	AT2G46790	NO
ZTL	AT5G57360	YES
ELF3	AT2G26930	YES
ELF4	AT2G40080	YES
LUX	AT3G46640	YES
FKF1	AT1G68050	YES
GI	AT1G22770	YES
FBH1	AT1G35460	YES
FBH2	AT4G09180	YES
FBH3	AT1G51140	YES
FBH4	AT2G42280	YES
CDF1	AT5G62430	YES
CDF2	AT5G39660	YES
CDF3	AT3G47500	YES
CDF4	AT2G34140	NO
PHYA	AT1G09570	YES
PHYB	AT2G18790	YES
PHYC	AT5G35840	YES
PHYD	AT4G16250	YES
PHYE	AT4G18130	YES
PIF1	AT2G20180	YES
PIF3	AT1G09530	YES
PIF4	AT2G43010	YES
PIF5	AT3G59060	YES
SPA1	AT2G46340	YES
SPA2	AT4G11110	YES
SPA3	AT3G15354	YES
SPA4	AT1G53090	YES
COP1	AT2G32950	YES
HOS1	AT2G39810	YES
PHL	AT1G672390	NO
CRY1	AT4G08920	YES
CRY2/FHA	AT1G04400	YES
NF-YA1	NF5G12840	NO
NF-YA2	AT3G05690	YES
NF-YA3	AT1G72830	YES
NF-YA4	AT2G34720	YES
NF-YA5	AT1G54160	YES
NF-YA6	AT3G14020	YES
NF-YA7	AT1G30500	YES
NF-YA8	AT1G17590	YES

NF-YA9	AT3G20910	YES
NF-YA10	AT5G06510	YES
NF-YB1	AT2G38880	YES
NF-YB2	AT5G47640	YES
NF-YB3	AT4G14540	YES
NF-YB4	AT1G09030	NO
NF-YB5	AT2G47810	NO
NF-YB6	AT5G47670	NO
NF-YB7	AT2G13570	NO
NF-YB8	AT2G37060	YES
NF-YB9	AT1G21970	NO
NF-YB10	AT3G53340	YES
NF-YB11	AT2G27470	NO
NF-YB12	AT5G08190	YES
NF-YB13	AT5G23090	YES
NF-YC1	AT3G48590	YES
NF-YC2	AT1G56170	YES
NF-YC3	AT1G54830	YES
NFYC4	AT5G63470	YES
NF-YC5	AT5G50490	NO
NF-YC6	AT5G50480	NO
NF-YC8	AT5G27910	NO
NF-YC9	AT1G08970	YES
NF-YC10	AT1G07980	YES
NF-YC11	AT3G12480	YES
NF-YC12	AT5G38140	YES
NF-YC13	AT5G43250	YES
AS1	AT2G37630	YES
CIB1	AT4G34530	YES
CIB2	AT5G48560	YES
CIB3	AT3G07340	YES
CIB4	AT1G10120	YES
CIB5	AT1G26260	YES
CO	AT5G15840	YES
SVP	AT2G22540	YES
FLM/MAF1	AT1G77080	YES
MAF2	AT5G65050	YES
MAF3	AT5G65060	YES
MAF4	AT5G65070	NO
MAF5	AT5G65080	NO
FLC	AT5G10140	YES
VIN3	AT5G57380	NO
CLF	AT2G23380	YES
VRN2	AT4G16845	YES
VIL1/VRN5	AT3G24440	YES
VIL2/VEL1	AT4G30200	YES
VIL3/VEL2	AT2G18880	YES

ELF7	AT1G79730	YES
VIP1	AT1G43700	YES
VIP2	AT5G59710	YES
VIP3	AT4G29830	YES
VIP4	AT5G61150	YES
VIP5	AT1G61040	YES
VIP6/ELF8	AT2G06210	YES
CDC73	AT3G22590	YES
HUB1	AT2G44950	YES
HUB2	AT1G55250	YES
ARP4	AT1G18450	YES
PIE1	AT3G12810	YES
ARP6	AT3G33520	YES
FRI	AT4G00650	YES
SUF4	AT1G30970	YES
ATXR7	AT5G42400	YES
ATX1	AT2G31650	YES
ATX2	AT1G05830	YES
ASH2R	AT1G51450	YES
WDR5a	AT3G49660	YES
EFS	AT1G77300	YES
LHP1	AT5G17690	YES
HUA1	AT3G12680	YES
HUA2	AT5G23150	YES
FLX	AT3G49725	YES
FCA	AT4G16280	YES
FPA	AT2G43410	YES
FY	AT5G13480	YES
FVE	AT2G19520	YES
FWA	AT4G25530	NO
FD	at4g35900	NO
14-3-3	AT1G22290	NO
14-3-3	AT1G22300	YES
14-3-3	AT1G26480	NO
14-3-3	AT1G34760	YES
14-3-3	AT1G78220	NO
14-3-3	AT2G42590	YES
SOC1	AT2G45660	YES
SEP3	AT1G24260	YES
FUL	AT5G60910	YES
AP1	AT1G69120	NO
LFY	AT5G61850	NO
AGL24	AT4G24540	YES
SPL3	AT2G33810	NO
SPL4	AT1G53160	YES
SPL5	AT3G15270	NO
SPL9	AT2G42200	YES

SPL15	AT3G57920	YES
GA1	AT4G02780	NO
GID1A	AT3G05120	YES
GID1B	AT3G63010	YES
GID1C	AT5G27320	YES
GNC	AT5G56860	YES
GNL	AT4G26150	YES
BOI	AT4G19700	YES
Della	AT1G14920	YES
TEM1	AT1G25560	YES
TEM2	AT1G68840	YES
FT	AT1G65480	NO
MFT	AT1G18100	NO
BFT	AT5G62040	NO
TSF	AT4G20370	NO
TFL	AT5G03840	NO
