# Supplementary material for: Oligosaccharide production and signaling correlate with delayed flowering in an Arabidopsis genotype grown and selected in high [CO2]
Source: PLoS One. 2023 Dec 28;18(12):e0287943. doi: 10.1371/journal.pone.0287943 (PMC10754469; doi:10.1371/journal.pone.0287943)
Supplement: S5 Table — (PDF) [file pone.0287943.s007.pdf]

S5 Table. Nutrient solution used by Ward lab (protocol from Duke Phytotron) Approximately ½ strength Hoagland's (A & B are mixed together)

Chemical	Stock solution (grams per L)	Working solution (ppm)	
	"A" solution		
Calcium nitrate $5[Ca(NO_3)_2]NH_4NO_3 \cdot 10H_2O$	277.7	Ca<br>N	103.00<br>86.39
Sequestrene 330 Fe DTPA (Sprint 138, BASF, USA)	15.0	Fe	3.00
	"B" solution		
Potassium nitrate KNO_3	152.02	K<br>N	117.58<br>42.20
Magnesium sulphate $MgSO_4 \cdot 7H_2O$	126.65	Mg<br>S	24.98<br>32.95
Zinc sulphate $ZnSO_4 \cdot 7H_2O$	0.286	Zn<br>S	0.13<br>0.064
Manganous sulphate $MnSO_4 \cdot 7H_2O$	0.195	Mn<br>S	0.127<br>0.074
Copper sulphate $CuSO_4 \cdot 5H_2O$	0.021	Cu<br>S	0.011<br>0.005
Boric acid H_3BO_3	0.725	B	0.254
Molybdic acid $MoO_3 \cdot 2H_2O$	0.005	Mo	0.005
Anhydrous Molybdic acid MoO_3	0.004	Mo	0.004
Potassium chloride KCl	3.154	K<br>Cl	3.31<br>3.0
Ammonium phosphate $NH_4H_2PO_4$	59.03	P<br>N	31.79<br>14.38
*Sodium Hydroxide NaOH		Na	

1. To make solution "A" and solution "B" add all "A" or "B" ingredients to 1L DI H_2O, then add an additional 1L DI H_2O. This keeps iron in solution "A" from precipitating.
2. Add 1 part "A" and 1 part "B" stock solution to 250 parts water.
3. *Adjust pH to 6.0 using NaOH.
